# The association between urinary bisphenol A levels and nonalcoholic fatty liver disease in Korean adults: Korean National Environmental Health Survey (KoNEHS) 2015-2017

**DOI:** 10.1186/s12199-021-01010-7

**Published:** 2021-09-14

**Authors:** Sang Joon An, Eun-Jung Yang, Subin Oh, Kyong Jin Park, Taehyen Kim, Yeon-pyo Hong, Yun-Jung Yang

**Affiliations:** 1 Department of Neurology, Catholic Kwandong University International St Mary’s Hospital, Incheon, 22711 Republic of Korea; 2 Department of Plastic and Reconstructive Surgery, Yonsei University College of Medicine, Seoul, 03722 Republic of Korea; 3 College of Medicine, Catholic Kwandong University, Gangneung-si, Gangwon-do 25601 Republic of Korea; 4 Department of Preventive Medicine, College of Medicine, Chung-Ang University, Seoul, 06974 Republic of Korea; 5 Institute of Biomedical Science, Catholic Kwandong University International St. Mary’s Hospital, Incheon, 22711 Republic of Korea

**Keywords:** Bisphenol A, Hepatic steatosis index, Nonalcoholic fatty liver disease, Korean adults, Korean national environmental health survey

## Abstract

**Background:**

Nonalcoholic fatty liver disease (NAFLD) is becoming a global health problem. Bisphenol A (BPA), one of most widely used environmental chemicals, is suspected to be a contributor to the development NAFLD. This study was performed to examine the relationship between human BPA levels and risk of NAFLD.

**Methods:**

The data (*n* = 3476 adults: 1474 men and 2002 women) used in this study were obtained from the Korean National Environmental Health Survey III (2015-2017). BPA levels were measured in urine samples. NAFLD was defined using hepatic steatosis index after exclusion of other causes of hepatic diseases.

**Results:**

There was a significant linear relationship between the elevated urinary BPA concentrations and risk of NAFLD. In a univariate analysis, odds ratio (OR) of the highest quartile of urinary BPA level was 1.47 [95% confidence interval (CI) 1.11-1.94] compared to the lowest quartile. After adjusted with covariates, the ORs for NAFLD in the third and fourth quartiles were 1.31 [95% CI 1.03-1.67] and 1.32 [95% CI 1.03–1.70], respectively.

**Conclusions:**

Urinary BPA levels are positively associated with the risk of NAFLD in adults. Further experimental studies are needed to understand the molecular mechanisms of BPA on NAFLD prevalence.

## Introduction

Nonalcoholic fatty liver (NAFLD) characterized by a fat accumulation of more than 5% in the liver without excessive alcohol use, viral hepatitis, and other liver diseases [1]. It encompassed a wide range of conditions from nonalcoholic fatty liver to more severe form, nonalcoholic steatohepatitis [2]. It is expected to be a primary cause of hepatocellular carcinoma and end-stage liver disease in the next few decades [3, 4]. The prevalence of NAFLD has been on the rise over recent decades and is estimated to be 25.24%, with 24.13% in the United States of America (USA) and 27.37% in Asia [5]. Although Western dietary consumption and inactive lifestyle contributed to the increasing rate of NAFLD [6], environmental chemicals which are frequently used in everyday material, can be the risk factor for NAFLD [7, 8].

Bisphenol A (BPA), one of the most widely used chemicals, consisted of two phenol rings attached by a methyl bridge, with two methyl groups [9]. It is utilized in the production of polycarbonate plastic and epoxy resin that are widely used in various applications including food packaging, beverage bottle, baby bottle, and paint [9, 10]. Because of the broad application, human can be exposed to BPA in their daily life and BPA is detected in over 90% of the humans [11, 12].

BPA is known to have estrogenic property by binding to the estrogenic receptors [9]. Many studies have reported the harmful health effects of BPA, including cancer, reproductive and development system, and neurological disorders [9, 13, 14]. Recently, epidemiological studies suggested the association between internal BPA levels and the risk of obesity and metabolic disorders, including insulin resistance, type 2 diabetes mellitus, and hyperlipidemia [15–17]. It might be due to the upregulation of lipogenic enzymes and transcription factors such as liver X receptor, the sterol regulatory element binding protein-1c, and the carbohydrate responsive element binding protein [18] and the elevation of oxidative stress [19]. BPA is associated with the weight gain, fat accumulation, and insulin resistance, thereby increasing the risk of NAFLD.

Previously, patients with histological diagnosis of NAFLD showed higher BPA levels in urine compared to the healthy subjects [7]. The positive association also showed between urinary BPA levels and NAFLD in USA population [20]. Nevertheless, the study from the USA included multiethnic populations; the majority of the race was non-Hispanic white [20]. Race/ethnicity and lifestyle could affect the NAFLD prevalence [1, 21]; however, the relationship between Asians and the risk of NAFLD prevalence has not been studied.

Therefore, this study was performed to evaluate the association between the urinary level of BPA and risk of NAFLD in Korean adults.

## Materials and methods

### Study population

This research data was taken from the Korean National Environmental Health Survey (KoNEHS) III (2015-2017). KoNEHS is conducted every 3 years from 2009 to understand the exposure levels of environmental chemicals, examine influential factors, and continuously investigate the factors of spatiotemporal distribution and changes in the Korean population. The third stage of KoNEHS was stratified into regional administrative and coastal regions and then classified based on the age, sex, and geographical regions socio-economic status. A total of 233 sampling units were randomly selected. After that, 15 households were selected using the systemic extraction method, and about 15 people were surveyed in each sample area [22].

**Fig. 1:**
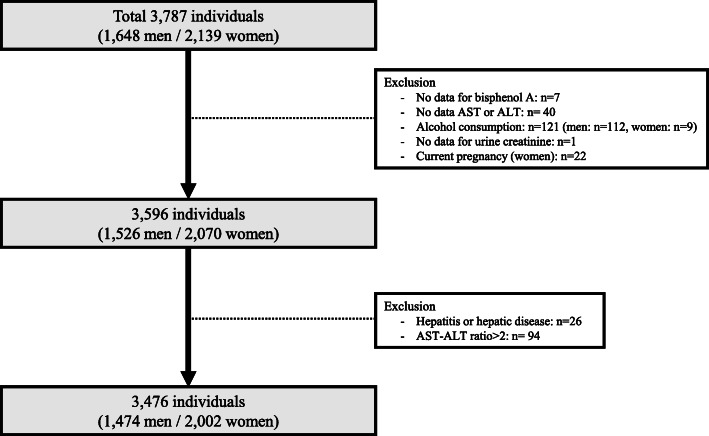
Study population in the present study obtained from the Korean National Environmental Health Survey III (2015-2017)

A total number of 3787 individuals (1648 men and 2139 women) with an age of ≥ 18 years underwent the questionnaires, anthropometric measurements, and collected urine and blood to analyze the levels of environmental chemicals. Three hundred eleven were excluded because of missing data for urinary BPA levels (*n* = 7), one of alanine aminotransferase (ALT) or aspartate aminotransferase (AST) levels (*n* = 40), and urine creatinine level (*n* = 1); those with significant alcohol consumption (*n* = 121; who consumed alcohol 3 times more than a week and 7-9 cups per time in men (*n* = 112) and who consumed alcohol 3 times more than a week and 5-6 cups per time in women (*n* = 19)); those with current pregnant women (*n* = 22); those with hepatitis or hepatic disease (*n* = 26); and those with AST/ALT ratio > 2 (*n* = 94). Finally, 3476 participants (1474 men and 2002 women) were included in this analysis (Fig. 1).

### Data collection and diagnosis

Demographic and lifestyle characteristics, including age, sex, drinking and smoking status, physical activity, monthly household income, education level, marriage, and medication taking were surveyed through face-to-face interviews. Based on their response, some variables were categorized as follows: education (less than high school graduate, high school graduate, and college graduate or higher), smoking (never, former, and current), physical activity levels (no, moderate, and vigorous), monthly household income (< 2, 2-3, 3-5, and > 5 million Korean won), and marital status (single, married, and divorced/separated). Drinking status was reclassified as never, former, and current according to the questionnaires: did not drink at all (never), had a drinking experience but did not drink at all in the last year (former), and had a drinking experience and drink more than once a week (current).

Participants with diagnosis of hepatitis or hepatic steatosis and who were presently under treatment or taking drugs were regarded as one that has the hepatic disease. Hypertension was defined by self-reporting a history of hypertension or taking antihypertensive medications. Diabetes mellitus (DM) was defined by self-reporting a history of DM or taking anti-diabetic medications. Hyperlipidemia was defined as a self-reported history of hyperlipidemia, use of anti-hyperlipidemic medication, high-density lipoprotein cholesterol ≤ 40 mg/dL, and triglyceride (TG) ≥ 240. Body mass index (BMI) was calculated as the body weight (kg) divided by height squared (m^2^).

### Urinary bisphenol A concentrations

Spot urine samples were collected from participants and stored at 0-4 °C immediately, and subsequently frozen at −20 °C according to the guideline of National Institute of Environmental Research [23]. Urinary BPA were measured using ultra-performance liquid chromatography-mass spectrometry (Xevo TQ-S, Waters, Milford, MA, USA) [24]. Values below the detection limit were divided by the square root of 2.

### Laboratory evaluations

Blood samples were collected from participants at the same time with the urine samples. The samples were centrifuged at 3500 rpm to separate the serum, and stored at 0-4 °C. The separated serum was aliquoted and frozen at −20 °C according to the guideline of National Institute of Environmental Research [23]. AST, ALT, and gamma-glutamyl transpeptidase (γ-GTP) were measured in serum samples [25]. The reference values were AST < 34U/L., ALT 10 - 49U/L, and γ-GTP < 73 U/L for men and γ-GTP < 38 U/L for women.

### NAFLD evaluations

The hepatic steatosis index (HSI) is an efficient non-invasive biomarker for NAFLD [26]. The variables in the HSI formula were levels of ALT, AST, BMI, sex, and presence of DM. Subjects were categorized into NAFLD and non-NAFLD using the published cut-off value of 36.

$$ \mathrm{HSI}=8\times \frac{\mathrm{ALT}}{\mathrm{AST}} ratio+ BMI\ \left(+2, if\ diabetes\ mellitues;+2, if\ female\right) $$

Since elevated AST, ALT, and γ-GTP were also used for NAFLD assessment [27–29], we defined abnormal AST, ALT, and γ-GTP based on the reference value.

### Statistical analysis

The average and standard error (continuous variables), and frequency (categorical variables) were provided in non-NAFLD and NAFLD based on the hepatic steatosis index score. Sample weights were included in order to reconstruct the data at the level of the entire population of Korea. The log transformation was performed because the distribution of urinary BPA concentrations was skewed to the right. BPA concentrations were categorized into quartiles based on the weighted sample distribution. The weighted mean (continuous variables) and weighted frequencies (categorical variables) in general characteristics were described by the BPA quartiles. The general characteristics were compared using the *t* test and an analysis of variance (continuous variable) or *χ*^2^ test (categorical variables).

A multivariate logistic regression analysis was performed to evaluate the relationship between urinary BPA levels and NAFLD. Covariates of age, sex, drinking, smoking, physical activity, monthly household income, education, marriage, and urine creatinine were included in the regression model. Because BMI is used in the HSI formula, it is not included in multivariate analysis as an independent variable to avoid collinearity. In this formula, 2 points was added in female to adjust the lower BMI in women compared to men. Also, because DM was an independent risk factor of NAFLD, 2 point was added in the subject with DM [26]. Thus, sex and DM were used as independent variables in multivariate analysis. Data analyses were performed using STATA (version 16.0 StataCorp LP College Station, TX, USA). *P* values < 0.05 were considered significant.

## Results

### General characteristics of study population

The general characteristic of samples was shown in Tables 1 and 2. Respondents were divided into two categories, non-NAFLD and NAFLD, based on the HSI (Table 1). Of 3476 participants, 2561 (73.68%) had HSI value under 36, and 915 (26.32%) had HSI value over 36 or higher. The mean age was 52.67 years old in non-NAFLD and 53.78 years old in NAFLD. Drinking status, physical activity, monthly income, education, and marital status was significantly different between non-NAFLD and NAFLD group (*p* = 0.018, *p* = 0.005, *p* = 0.037, *p* < 0.001, and *p* = 0.043, respectively). Participants in the NAFLD group had significantly higher levels in AST, ALT, γ-GTP, and BMI (all *p* < 0.001).

**Table 1:** General characteristics of study participants according to hepatic steatosis index score

	Total (*n* = 3476)	non-NAFLD (*n* = 2561)	NAFLD (*n* = 915)	*p* value
Age, year	52.96 ± 0.25	52.67 ± 0.29	53.78 ± 0.46	0.090
Gender (%, men)	1474 (42.41)	1070 (41.78)	404 (44.15)	0.213
BMI, kg/m^2^	24.61 ± 0.05	23.23 ± 0.04	28.47±0.10	< 0.001
Drinking status, *n* (%)				0.018
Never	715 (20.57)	502 (19.60)	213 (23.28)	
Former	439 (12.63)	314 (12.26)	125 (13.66)	
Current	2322 (66.80)	1745 (68.14)	577 (63.06)	
Smoking status, *n* (%)				0.727
Never	2278 (65.54)	1667 (65.48)	602 (65.68)	
Former	679 (19.53)	507 (19.80)	172 (18.80)	
Current	519 (14.93)	377 (14.72)	142 (15.52)	
Physical activity, *n* (%)				0.005
No	1888 (54.32)	1350 (52.71)	538 (58.80)	
Moderate	274 (7.88)	205 (8.00)	69 (7.54)	
Vigorous	1314 (37.80)	1006 (39.28)	308 (33.66)	
Monthly household income, *n* (%)				0.037
< 2 million Korean Won	1308 (37.63)	944 (36.86)	364 (39.78)	
2-3 million Korean Won	733 (21.09)	523 (20.42)	210 (22.95)	
3-5 million Korean Won	873 (25.12)	662 (25.93)	209 (22.84)	
> 5 million Korean Won	562 (16.17)	430 (16.79)	132 (14.43)	
Education, *n* (%)				< 0.001
< High school	1172 (33.72)	800 (31.24)	372 (40.66)	
High school	1220 (35.10)	941 (36.74)	279 (30.49)	
College or higher	10.84 (31.19)	820 (32.02)	264 (28.85)	
Marital status, *n* (%)				0.043
Single	397 (11.42)	310 (12.10)	87 (9.51)	
Married	2681 (77.13)	1971 (76.96)	710 (77.60)	
Divorced or separated	398 (11.45)	280 (10.93)	118 (12.90)	
AST	25.97 ± 0.17	24.95 ± 0.18	28.84 ± 0.41	< 0.001
ALT	24.86 ± 0.26	20.82 ± 0.18	36.17 ± 0.74	< 0.001
γ-GTP	29.78 ± 0.57	26.00 ± 0.61	40.36 ± 1.29	< 0.001
Comorbidity, *n* (%)				
Hypertension	798 (22.96)	474 (18.51)	324 (35.41)	< 0.001
Diabetes mellitus	309 (8.89)	146 (5.70)	163 (17.81)	< 0.001
Hyperlipidemia	1211 (34.84)	734 (28.66)	477 (52.13)	< 0.001

*NAFLD* nonalcoholic fatty liver disease, *BMI* body mass index, *AST* aspartate aminotransferase, *ALT* alanine aminotransferase, *γ-GTP* gamma-glutamyl transpeptidase

**Table 2:** General characteristics of study participants according to urinary bisphenol A concentrations

	Quartile 1 (*n* = 888)	Quartile 2 (*n* = 880)	Quartile 3 (*n* = 860)	Quartile 4 (*n* = 848)	*P* value
Age (year)	47.60 ± 0.96	47.23 ± 0.93	47.08 ± 0.88	45.82 ± 0.74	0.135
Gender (%, men)	43.98 ± 1.87	45.03 ± 1.83	50.84 ± 2.24	56.02 ± 1.91	< 0.001
BMI (kg/m^2^)	23.87 ± 0.14	24.17 ± 0.16	24.61 ± 0.19	24.73 ± 0.17	< 0.001
Drinking status (%)					0.056
Never	17.13 ± 1.80	17.37 ± 1.73	14.72 ± 1.51	13.71 ± 1.46	
Former	11.64 ± 1.34	12.83 ± 1.59	10.27 ± 1.59	10.91 ± 1.38	
Current	71.21 ± 2.08	69.77 ± 2.11	75.00 ± 1.95	75.37 ± 1.98	
Smoking status (%)					< 0.001
Never	70.06 ± 1.71	65.52 ± 1.91	59.79 ± 2.53	58.71 ± 2.42	
Former	16.16 ± 1.51	18.49 ± 1.67	20.25 ± 1.80	18.24 ± 1.83	
Current	13.77 ± 1.76	15.97 ± 2.09	20.24 ± 1.82	23.03 ± 2.10	
Physical activity (%)					0.099
No	52.75 ± 2.34	54.60 ± 2.46	54.01 ± 2.34	60.38 ± 2.05	
Moderate	9.68 ± 1.42	6.44 ± 1.07	7.71 ± 1.19	5.79 ± 0.96	
Vigorous	37.56 ± 2.61	38.95 ± 2.42	38.27 ± 2.16	33.82 ± 2.15	
Monthly household income (%)					0.943
< 2 million Korean Won	28.53 ± 2.63	27.09 ± 2.42	25.90 ± 1.89	28.94 ± 2.66	
2-3 million Korean Won	19.55 ± 2.32	25.18 ± 2.14	22.59 ± 2.14	20.34 ± 1.97	
3-5 million Korean Won	30.38 ± 2.72	25.97 ± 2.53	30.14 ± 2.19	29.79 ± 2.72	
> 5 million Korean Won	21.52 ± 2.64	21.75 ± 2.34	21.35 ± 2.33	20.91 ± 2.14	
Education (%)					0.996
< High school	22.41 ± 2.26	20.30 ± 1.90	21.73 ± 1.92	20.25 ± 1.82	
High school	35.01 ± 2.13	38.62 ± 2.07	38.93 ± 2.64	38.25 ± 2.32	
College and more	42.56 ± 2.71	41.07 ± 2.21	39.33 ± 2.66	41.49 ± 2.53	
Marital status (%)					0.797
Single	21.02 ± 2.29	21.65 ± 2.18	20.27 ± 2.25	22.42 ± 2.14	
Married	71.13 ± 2.35	69.49 ± 2.39	71.26 ± 2.33	69.69 ± 2.23	
Divorced or separated	7.84 ± 1.16	8.85 ± 1.26	8.45 ± 1.32	7.87 ± 1.13	
AST	25.57 ± 0.35	24.79 ± 0.32	25.90 ± 0.82	25.42 ± 0.45	0.048
ALT	24.70 ± 0.62	24.09 ± 0.67	25.91 ± 1.19	26.26 ± 0.72	0.729
γ-GTP	28.18 ± 1.25	28.49 ± 1.25	32.20 ± 1.88	32.57 ± 1.87	0.015
Comorbidity (%)					
Hypertension	16.78 ± 1.21	17.06 ± 1.62	17.29 ± 1.40	14.40 ± 1.54	0.257
Diabetes mellitus	6.44 ± 0.98	4.65 ± 0.63	6.02 ± 1.13	7.39 ± 1.10	0.359
Hyperlipidemia	27.92 ± 2.11	30.54 ± 2.00	31.41 ± 2.56	34.44 ± 2.12	0.025

*BMI* body mass index, *AST* aspartate aminotransferase, *ALT* alanine aminotransferase, *γ-GTP*, gamma-glutamyl transpeptidase. Data were shown as the weighted mean or frequency ± standard errors as appropriated

Table 2 indicated the general characteristics of study participants according to urinary BPA concentration. BPA concentrations were log-transformed because of the skewness. The transformed BPA level was classified into quartiles based on the weighted sample distribution. The range of BPA concentration (μg/L) is shown as follows: min-0.50 in the first quartile, 0.50-1.26 in the second quartile, 1.26-2.67 in the third quartile, and 2.67-max in the fourth quartile. Gender, BMI, smoking status, AST, γ-GTP, and hyperlipidemia were found to be statistically significant among the levels of BPA (*p* < 0.001, *p* < 0.001, *p* < 0.001, *p *= 0.048, *p *= 0.015, and *p *= 0.025, respectively).

### Distribution of urinary BPA concentrations

The urinary BPA levels were shown in Table 3 between the non-NAFLD group and NAFLD group. The geometric mean (GM) concentration of BPA in NAFLD group (2.56 μg/L) was significantly higher than in non-NAFLD group (2.24 μg/L) (*p* = 0.001). The 25th, 50th, and 75th levels of urinary BPA were 0.48 μg/L, 1.21 μg/L, and 2.56 μg/L in non-NAFLD group, and 0.57 μg/L, 1.52 μg/L, and 2.96 μg/L in NAFLD group.

**Table 3:** Distributions of urinary bisphenol A levels (BPA, μg/L) in the study population

BPA conc. (μg/L)	Total	non-NAFLD	NAFLD	***P*** value
GM (GSE)	2.32 (0.07)	2.24 (0.08)	2.56 (0.15)	0.001
Percentile				
Min	0.05	0.05	0.05	
25th	0.5	0.48	0.57	
50th	1.26	1.21	1.52	
75th	2.66	2.56	2.96	
Max	111.31	111.31	86.78	

*NAFLD* nonalcoholic fatty liver disease, *GM* geometric mean, *GSE* geometric standard error

### Prevalence of NAFLD and abnormal ALT, AST, and γ-GTP according to urinary BPA levels

The number of NAFLD based on HSI, and abnormal ALT, AST, and γ-GTP were presented in Table 4. The prevalence of NAFLD based on HSI and abnormal ALT were increased in accordance with the increase of urinary BPA concentrations (*p* = 0.002 and *p* = 0.023, respectively). However, there were no significant relationships between the number of abnormal AST and γ-GTP with urinary BPA levels.

**Table 4:** Prevalence of NAFLD and abnormal ALT, AST, and γ-GTP activities according to urinary bisphenol A levels

		Quartile 1 (*n* = 888)	Quartile 2 (*n* = 880)	Quartile 3 (*n* = 860)	Quartile 4 (*n* = 848)	*P* value
NAFLD	Actual number	201	214	242	258	0.002
	Weighted frequency (95% CI)	21.83 (18.42-25.68)	22.14 (18.88-25.78)	25.55 (21.64-29.90)	29.18 (25.48-33.19)	
Abnormal ALT	Actual number	83	88	87	102	0.023
	Weighted frequency (95% CI)	10.41 (8.22-13.11)	12.27 (9.36-15.93)	11.25 (8.80-14.27)	15.34 (12.53-18.66)	
Abnormal AST	Actual number	110	82	91	10	0.872
	Weighted frequency (95% CI)	12.24 (9.62-15.45)	7.59 (5.67-10.09)	9.99 (8.03-12.35)	11.77 (9.45-14.58)	
Abnormal γ-GTP	Actual number	104	89	99	95	0.844
	Weighted frequency (95% CI)	10.86 (8.54-13.72)	9.29 (6.97-12.29)	12.05 (9.42-15.29)	10.31 (8.06-13.09)	

*NAFLD* nonalcoholic fatty liver disease, *AST* aspartate aminotransferase, *ALT* alanine aminotransferase, *γ-GTP* gamma-glutamyl transpeptidase

### The association between urinary BPA concentrations and NAFLD prevalence

Odds ratio (OR) and 95% CI for NAFLD based on HSI according to BPA levels were presented in Table 5. A logistic regression was conducted based on the BPA quartile, using the first quantile as the reference group.

**Table 5:** Multivariate analysis for NAFLD according to urinary bisphenol A levels

	Crude		Model 1		Model 2		Model 3	
	OR (95% CI)	***P*** value	OR (95% CI)	***P*** value	OR (95% CI)	***P*** value	OR (95% CI)	***P*** value
Quartile 1	1	0.002^*^	1	0.035^*^	1	0.003^*^	1	0.010^*^
Quartile 2	1.01 (0.74-1.39)	0.913	1.00 (0.73-1.37)	0.955	1.09 (0.87-1.36)	0.435	1.08 (0.85-1.36)	0.510
Quartile 3	1.22 (0.89-1.68)	0.203	1.16 (0.84-1.58)	0.346	1.28 (1.02-1.60)	0.032	1.31 (1.03-1.67)	0.025
Quartile 4	1.47 (1.11-1.94)	0.006	1.31 (0.99-1.75)	0.058	1.37 (1.08-1.73)	0.007	1.32 (1.03-1.70)	0.026

*NAFLD* nonalcoholic fatty liver disease, *OR* odds ratio, *CI* confidence interval

*p values were shown the test of trend of odds

Crude: Hepatic steatosis index, bisphenol A

Model 1: Crude + age, gender, creatinine

Model 2: Model 1 + smoking, drinking, exercise, marital status, education, income

Model 3: Model 2 + hypertension, diabetes mellitus, hyperlipidemia

In a crude model, the fourth quartile showed a significantly higher OR of HSI (1.47 [95% CI 1.11-1.94]). When adjusting age, gender and urine creatinine (model 1), OR in the fourth quartile was 1.31 (95% CI 0.99-1.75). Hence, there was no significant association.

With an additional adjustment of the socio-economic values (model 2), the third and fourth quartiles showed higher ORs of NAFLD by HSI compared to the first quartile (1.28 [95% CI 1.02-1.60] and 1.37 [95% CI 1.08-1.73], respectively).

Further adjusting comorbidities such as hypertension, DM, and hyperlipidemia (model 3), ORs in the third and fourth quartiles were higher than in reference group (1.31 [95% CI 1.03-1.67] and 1.32 [95% CI 1.03-1.70] respectively).

Results of trend tests for the correlation between NAFLD and BPA were *p* < 0.05 regardless of adjustments (crude, model 1, model 2, and model 3).

## Discussion

This study was conducted to evaluate the association between urinary BPA level and NAFLD base on HSI score in the Korean population. The higher risk of NAFLD according to HSI was significantly associated with higher urinary BPA concentration. Exposure of BPA presented by urine BPA level linearly correlated with NAFLD occurrence in both unadjusted and adjusted models.

BPA is widely used in the synthesis of consumer products, including food and beverage containers, baby bottle, and dental sealants [9, 10]. Human are exposed to BPA through oral administration, inhalation and dermal absorption [30]. Once BPA enters to body, it binds to glucuronic acid and converts to BPA glucuronide in the liver and gut [9], and the BPA glucuronides are primarily excreted through urine [31]. Although some prospective studies used in both urine and blood samples to measure BPA levels [7, 32], urine samples were used to demonstrate the BPA exposure in population based studies because of the relatively ease to collect the biological samples compared to the blood.

BPA has been suggested that the positive relationship with obesity and metabolic disease in healthy adults [15, 16, 33]. Obesity is well-known factor in liver abnormalities, including NAFLD [34] that is characterized by an increase of hepatic TG content with or without inflammation and fibrosis [1, 2]. Recent epidemiologic studies have showed an association between urinary BPA levels and the risk of NAFLD [7, 20]. It seems that BPA could promote lipid accumulation in the liver as well as obesity.

Experimental studies have suggested the underlying mechanism for the hepatic lipid accumulation due to BPA exposure. BPA-treated mice showed the increase of lipid accumulation in the liver through the activation of lipogenic enzymes, including acetyl-CoA carboxylase, fatty acid synthase, stearoyl-CoA desaturase-1, and sterol regulatory element binding factor 1 [18, 35]. In addition, the elevation of inflammation might be closely associated with the appearance and progression of liver disease [7, 19, 36]. Thus, BPA could induce hepatic steatosis through modulated by de novo lipogenesis and inflammation.

Previously, BPA levels in urine and blood in NAFLD patients was significantly higher than healthy subjects [7]. In US population based study, the positive association also showed between urinary BPA concentrations and the prevalence of NAFLD [20]. Similar with the previous studies, higher levels of urinary BPA were associated with NAFLD prevalence in Korean adults. Although BPA is metabolized and excreted over 90% within 24 h after administration [37], most people can be exposed to BPA through their life. In addition, internal BPA levels can vary depending on the lifestyle, diet, and living environment [38]. Thus, further study is needed on whether the reduction of BPA exposure can decrease the incidence of NAFLD.

In addition, the prevalence of abnormal ALT was increased according to increase of BPA levels, but not in AST and γ-GTP. Similar with our result, serum ALT levels was relatively higher in NAFLD patients than healthy people [28, 39]. Because the increase of hepatic lipid accumulation can induce the toxic effects on hepatocytes [40], NAFLD may induce the elevation of liver enzymes, including ALT, AST, and GGT. It seems that exposure to BPA might increase the risk of liver damages.

To the best of our knowledge, this is the first study to establish the association between urinary BPA level and NAFLD prevalence in the Korean population. In addition, because study was performed from the data that can represent the national population, these results could generalize to the population. However, there remains some limitation. First, HSI score is used to define NAFLD. Liver biopsy and abdominal ultrasound might be more appropriate to find liver steatosis. However, those are not feasible in population based large-scale study because of the invasiveness and cost issue. HSI is a noninvasive tool to predict the presence of NAFLD using anthropometric and laboratory parameters, including BMI, AST, and ALT [26]. HSI score with < 36 or > 36 had 93.1% sensitivity to rule-out the absence of NAFLD and 92.4% specificity to detect the presence of NAFLD. Nevertheless, there is still a problem with classification errors, because 30 ≤ HSI ≤ 36 was considered as the non-NAFLD group. However, it is inevitable to statistical analysis. Second, the amount of alcohol consumption (g/day) could not estimate because KoNEHS offered the drinking times in the last month and the number of glass per times. In this study, men who consumed alcohol 3 times more than a week and 7-9 glasses per time, and women who consumed alcohol 3 times more than a week and 5-6 glasses per time were defined as heavy drinker by referring to previous study [41]. Third, it could not evaluate the causal relationship between urinary BPA level and NAFLD because of a cross-sectional analysis. Additional studies are needed to determine the contribution of BPA on NAFLD prevalence. Fourth, BPA levels were measured using spot urine samples. After administration, most of BPA is eliminated within 24 h via urine [37]; thus, temporal variation of BPA levels in subjects can be raised. Nevertheless, KoNEHS randomly collected the spot urine sample from a large population according to the survey guideline [23]. It may reflect the average BPA levels in population.

In conclusion, the result of this study based on a representative Korean population indicated that there is positive association between urine BPA concentration reflecting BPA exposure and risk of increasing NAFLD. As BPA has been observed to be associated with an increased prevalence of NAFLD, it would be desirable to reduce BPA exposure.

## Data Availability

Our investigation was based on KoNEHS cycle 3 (2015–2017), which was approved by the institutional review board of the National Institute of Environmental Research in Korea (NIER-2016-BR-003-01, NIER-2016-BR-003-03). All participants provided written informed consent. The data presented in this study are available on request from the corresponding author. In this case, the researcher receives data that does not include identifiable IDs and/or regional code variables in combination with personal information and other data.
